# The NRF2 Signaling Network Defines Clinical Biomarkers and Therapeutic Opportunity in Friedreich’s Ataxia

**DOI:** 10.3390/ijms21030916

**Published:** 2020-01-30

**Authors:** Piergiorgio La Rosa, Enrico Silvio Bertini, Fiorella Piemonte

**Affiliations:** Unit of Muscular and Neurodegenerative Diseases, Bambino Gesù Children’s Hospital, IRCCS, 00146 Rome, Italy; piergiorgio.larosa@opbg.net (P.L.R.); enricosilvio.bertini@opbg.net (E.S.B.)

**Keywords:** Friedreich’s ataxia, NRF2, redox active drugs, frataxin, neurodegenerative diseases

## Abstract

Friedreich’s ataxia (FA) is a trinucleotide repeats expansion neurodegenerative disorder, for which no cure or approved therapies are present. In most cases, GAA trinucleotide repetitions in the first intron of the *FXN* gene are the genetic trigger of FA, determining a strong reduction of frataxin, a mitochondrial protein involved in iron homeostasis. Frataxin depletion impairs iron–sulfur cluster biosynthesis and determines iron accumulation in the mitochondria. Mounting evidence suggests that these defects increase oxidative stress susceptibility and reactive oxygen species production in FA, where the pathologic picture is worsened by a defective regulation of the expression and signaling pathway modulation of the transcription factor NF-E2 p45-related factor 2 (NRF2), one of the fundamental mediators of the cellular antioxidant response. NRF2 protein downregulation and impairment of its nuclear translocation can compromise the adequate cellular response to the frataxin depletion-dependent redox imbalance. As NRF2 stability, expression, and activation can be modulated by diverse natural and synthetic compounds, efforts have been made in recent years to understand if regulating NRF2 signaling might ameliorate the pathologic defects in FA. Here we provide an analysis of the pharmaceutical interventions aimed at restoring the NRF2 signaling network in FA, elucidating specific biomarkers useful for monitoring therapeutic effectiveness, and developing new therapeutic tools.

## 1. Introduction

In the cellular environment, superoxide anion (O_2_^•−^), hydrogen peroxide (H_2_O_2_), and the hydroxyl radical (^•^OH) arise from the incomplete reduction of oxygen [1]. These molecules, collectively known as reactive oxygen species (ROS), exert discrete biological properties due to differences in lipid solubility, half-life, and chemical responsiveness [2], which confer reactivity to specific biological targets and the evolution-selected activation of signal transduction mechanisms involved in DNA, lipid, and protein damage/repair [1,2,3,4]. Indeed, a tight control of ROS production and elimination is pursued in the cell, to allow the activation of signaling cascades and to minimize the entity of adverse effects. This equilibrium is obtained by the constitutive production of the cellular antioxidant machinery and from non-enzymatic compounds. However, when this homeostasis cannot be maintained and oxidative stress (i.e., the pathologic condition caused by the imbalance between ROS production and antioxidant response) occurs, the cell reacts by upregulating the synthesis of specific antioxidants and enzymes, through the modulation of different transcriptional factors, whose master regulator is represented by NF-E2 p45-related factor 2 (NRF2) [5,6]. Beside its role in redox maintenance of homeostasis, NRF2 functions span multiple cellular processes, including survival, metabolic and protein homeostasis, inflammation, and regulation of cell proliferation/differentiation [7,8,9,10,11]. Thus, it is not surprising that the deregulation of NRF2 expression or activity has been found in many diseases in which oxidative stress represents a typical pathologic feature, including cancer [12], multiple sclerosis (MS) [9], Alzheimer’s disease (AD) [13], Parkinson’s disease (PD) [13], and amyotrophic lateral sclerosis (ASL) [14]. It is important to note that, especially in regard of neurodegenerative diseases, efforts to re-establish a proper NRF2 signaling network led to improvements of neurological phenotypes [15,16].

Among the neurodegenerative diseases characterized by redox imbalance, Friedreich’s ataxia (FA) is an autosomal recessive disease representing the most common inherited form of ataxia. Although high variability occurs in the population, 1:50,000 individuals are estimated to be affected by the pathology, while 1:110 is carrier of the gene dysfunction [17,18]. In 95% of cases, the genetic dysfunction responsible for the disease is caused by 200–1700 GAA trinucleotide repetitions inserted in the first intron of both alleles of *FXN* gene, while in 5% of patients one expanded allele is paired with a point mutation in the second one [19,20,21]. For this reason, FA is commonly included among the nucleotide repeats expansion disorders and, although aberrant RNA structures and toxic gain of function of the RNA or protein products are believed to be the pathogenic mechanism characterizing many of this heterogeneous group of pathologies [22], the lack of the mitochondrial protein frataxin, the product of *FXN* [17], represents the molecular determinant underlying the disease. In affected probands, *FXN* expression ranges between 5% and 35%, in normal individuals, while 50% of frataxin expression has been found in asymptomatic heterozygotes [23]. Different mechanisms have been proposed to explain the strong reduction of *FXN* expression: (i) the induction of GAA repeat-induced DNA triplexes or “sticky DNA” conformations that interfere with *FXN* gene transcription [24,25,26]; (ii) the formation of R-loop structures between the nascent *FXN* mRNA and the DNA template strand, determining RNA polymerase II pausing and transcription termination [27]; and (iii) the presence of repressive heterochromatic structures in the proximity of the GAA-repeated tract, which extends to the *FXN* promoter thereby causing a reduced initiation of the transcriptional process [28,29], partially exploited by histone deacetylase inhibitors [30,31]. 

**Figure 1:**
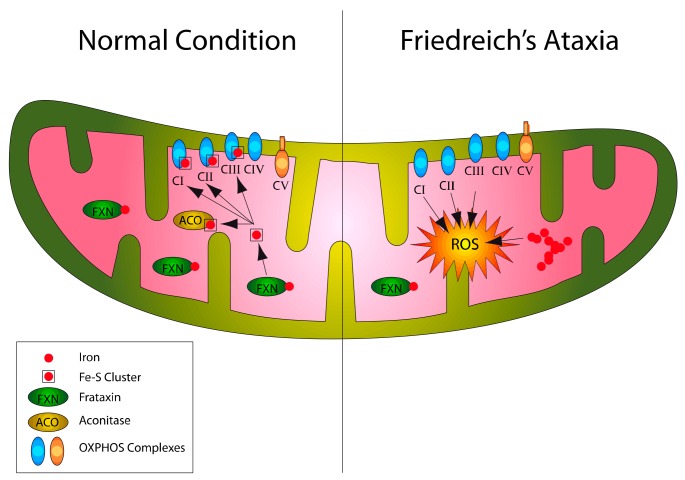
Overview of Friedreich’s ataxia deregulations following frataxin expression deficiency. Frataxin depletion determines iron accumulation in the mitochondrion, impairments of Fe–S proteins, increased reactive oxygen species (ROS) accumulation and oxidative stress.

Although, a number of frataxin functions related to iron transport and storage [32], mitochondrial biogenesis [33], regulation of apoptosis and ferroptosis [34,35], and antioxidant defenses [36] are only partly understood, is widely accepted that frataxinparticipates to the synthesis of iron–sulfur clusters (ISC) in the mitochondria [37]. As a consequence, frataxindeficiency has been reported to impair the activity of ISC-containing enzymes, such as the respiratory chain complexes I, II, III and aconitase [38,39], and those involved in the heme biosynthesis [40], and to determine mitochondrial iron accumulation [38,41]. The Fenton-mediated increase of superoxide anion and hydroxyl radicals resulting from a dysfunctional respiratory chain and iron accumulation has led to the hypothesis of increased oxidative stress in FA and enhancement of lipid peroxidation [42] (Figure 1). Although, some studies still question the role of oxidative stress in FA [43,44], oxidative stress-induced defects have been reported in yeast [45,46], drosophila [47,48], and mice models [49,50,51], and altered levels of redox markers have been found in blood [52,53,54] and fibroblasts [55] of patients.

To note, although increased oxidative stress should activate cellular antioxidant defense, on the contrary NRF2 and its signaling pathway are faulty in FA [56,57,58,59]. 

This review provides an overview of NRF2 signaling in FA, focusing on its impairment and the transcriptional defects of its target genes. We will analyze the pharmacological and experimental progress of researchers in restoring NRF2 expression and activity in this disease. Particular emphasis will be given to promising treatments able to rescue NRF2 activity in vitro and to highlight specific biomarkers belonging to NRF2 signaling network. 

## 2. NRF2 Signaling Network Overview and Impairments in FA

NRF2 is a transcription factor encoded by the *NFE2L2* gene in humans, belonging to basic leucine zipper (bZip) protein family, with a cap’n’collar (CNC) structure [60]. CNC proteins cannot bind to DNA as monomers [61], they dimerize in the nucleus with other proteins that share this structural moiety [62]. NRF2, in particular, is able to interact with CNC-bZip domain-containing family isoforms F, G, and K of small musculoaponeurotic fibrosarcoma (MAF) proteins [63,64], which become NRF2 obliged partners. The formation of NRF2/sMAF heterodimer allows the recognition of 16 specific base pair enhancer-acting DNA sequences known as antioxidant responsive elements (ARE) [65] that share similarity both with NRF2-binding motif and sMAF-recognized DNA sequences [5], found in about 250 genes [8]. Although two nuclear localization and one nuclear export signals modulate NRF2 cellular trafficking [66], under physiological conditions NRF2 is mainly cytoplasmic and only a basal transcription of ARE-containing genes is allowed. This is mediated by the NRF2 pool which escapes the fine-tuned degradation mechanism that limits the protein half-life to 15–40 min, depending on cell type [67]. After its synthesis, NRF2 interacts in the cytoplasm with homodimers of Kelch-like ECH-associated protein 1 (KEAP1), a ubiquitin ligase adaptor protein, that presents NRF2 to the ubiquitin E3 ligase complex Cullin-3 (CUL3)/RBX1 leading to its polyubiquitination and proteasomal degradation [68]. Alternatively, the kinase GSK3β can phosphorylate NRF2 in serine residues 335 and 338, allowing its recognition by the β transducin repeat containing E3 ubiquitin-protein ligase (βTrCP) and allowing NRF2 polyubiquitination and CUL3/RBX1-mediated degradation [6,67]. These negative regulations guarantee a low rate of ARE-containing genes transcription, as sMAF homodimers lacking the ability to activate gene transcription are more frequently produced in this condition [69]. At the same time, this regulation circuitry provides a basal transcription of ARE-containing genes, as a small fraction of NRF2 cellular content escapes its negative regulators. Indeed, discrete cellular cues are able to hijack NRF2 sequestration and degradation complexes, increasing NRF2 stability and allowing its nuclear migration. In the case of GSK3β-mediated degradation, the activation of specific ion channels, G protein-coupled receptors and tyrosine kinase growth factor receptors, leads to the induction of the PI3K/AKT pathway determining the inhibitory phosphorylation of GSK3β [70]. In the same way, the impairment of NRF2 interaction with KEAP1–CUL3/RBX1 complex determines NRF2 translocation into the nucleus. ROS-induced modification of KEAP1 critical cysteine residues [71], signal transduction mechanism-dependent NRF2 phosphorylation [72,73], and the direct p62-KEAP1 [74] or p21-NRF2 [75] interaction are responsible of NRF2 or KEAP1 conformational changes determining the inhibition of NRF2 degradation and triggering the increase of ARE-dependent transcription. 

In line with this principle, as has been observed in other neurodegenerative diseases where oxidative stress is increased [76], in FA the frataxin deficiency-induced oxidative stress should stabilize NRF2 and induce an up-regulation of ARE-dependent gene transcription. Nevertheless, NRF2 signaling is impaired in several FA cells and animal models [10,36,56,57,58,59,77]. The mechanisms underlying NRF2 deficiency in FA are still poorly understood, although, starting from the first decade of the 2000s some studies reported a reduced antioxidant defense as a consequence of frataxin depletion [78], while the over-expression of frataxinwas able to potentiate the antioxidant responses [79]. Some years later, a study reported on microarray analyses performed on the blood of 26 children with FA that confirmed a specific down-regulation of NRF2 target genes [36] and subsequent studies further showed impairments of NRF2 expression and nuclear translocation in FA fibroblasts [58] and in motor neuron-like cells [57]. The mechanism proposed in those studies highlighted an abnormal distribution of actin fibers causing a defective binding of the NRF2–KEAP1 complex and the failure of NRF2 nuclear translocation [58]. Beside this, defects in redox homeostasis and in the NRF2-dependent antioxidant response are reported in many other models of the disease, such as in the hearts of the muscle creatine kinase (MCK) conditional frataxin knockout mice [56], in dorsal root ganglia (DRG) neurons dissected from the YG8R mice [59] and in neural stem cells (NSCs) isolated from the knock-in knock-out (KIKO) FA mouse model [10]. These numerous reports strongly address therapeutic approaches aimed at re-establishing a functional NRF2 signaling in FA. 

## 3. Therapeutic Intervention in the Regulation of NRF2-Mediated Signaling Pathway

KEAP1 is rich of cysteines (Cys): 25 residues have been identified in the mouse and 27 in the human protein [80], while the human and mouse NRF2 display respectively six and seven Cys residues [81]. These amino acids are highly reactive and different compounds can bind to them by inducing conformational changes able to interfere with the NRF2–KEAP1–CUL3 complex, inhibiting NRF2 ubiquitination and inducing its activity [5]. Mediators of this regulation system are two binding motifs located in the Neh2 domain of NRF2, the ETGE and DLG motifs, that interact with the homodimer of KEAP1 in a “two site substrate recognition” [82]. KEAP1 homodimer is also capable of interaction with the CUL3-based E3 ligase complex, thereby mediating NRF2 polyubiquitination [83]. Upon oxidative stress or compound interaction, the cysteine residues of KEAP1 undergo a conformational modification that compromises their interaction with the low affinity DLG motif, while the ETGE motif remains associated to the inhibitory complex [84]. This does not determine the NRF2 release from KEAP1, however it impairs the NRF2 degradation, thus newly synthesized NRF2 may translocate to the nucleus and start the transcription of its target genes [85]. Therefore, it is evident how the process of covalent modification of KEAP1 sulfhydryl group-containing cysteine residues is critical in modulating NRF2 activity, and how different molecules, directed to specific cysteines, can alternatively modulate the redox function, also guaranteed by the high plasticity in the NRF2–KEAP1 chemical recognition. It is from the susceptibility of these different cysteine residues that the term “cysteine code” originates. NRF2 activation is induced, for instance, by the modification of Cys^151^ in mouse, while Cys^273^ and Cys^288^ are crucial for mediating the KEAP1-induced NRF2 repression in unstressed conditions [86]. The highly conserved Cys residues of NRF2 can participate in this regulation circuitry, having been proved essential to repress the KEAP1-dependent degradation and promote coactivator recruitment [81]. 

Given the complexity of this regulatory system, it is not surprising that a variety of chemicals, both natural and synthetic and very different in structure, are able to interfere with the NRF2–KEAP1 complex, although very few common properties are reflected among them [83,87]. The isothiocyanate sulphoraphane (SFN) was one of the first identified activators of NRF2 and one of the most potent [88,89]. The SFN-mediated NRF2 induction, through the interaction with the Cys^151^ of KEAP1, has beneficial effects in pre-clinical models and in the clinical treatment of neurological conditions [90,91]. In FA, in vitro SFN treatment partially rescues the cellular phenotypic defects in frataxin-silenced motor neuron-like cells [92], in neural stem cells isolated from the KIKO FA mouse model [10] and in FA fibroblasts [77]. Despite this, SFN is actually not adopted in FA clinical trials, probably because of its off-target activity [93] and the low blood–brain barrier permeability [94]. In the same way, the synthetic compound CAT-4001, although never tested in humans, has shown promising results in FA mice by improving mitochondrial biogenesis of isolated DRG neurons [95,96].

On the other hand, several antioxidant compounds (i.e., coenzyme Q10, vitamin E, idebenone, EPI-743, and MitoQ), sharing a similar chemical structure, have been tested in the treatment of patients with FA and in other mitochondrial diseases. These molecules display general lipophilic properties and act as potent antioxidants [97,98,99] inhibiting lipid peroxidation [100,101]. Although a direct interaction of quinone compounds with KEAP1 Cys residues has not yet been described, indirect proof has led to the proposal that these molecules are NRF2 inducers. In addition to their antioxidant activity: (i) coenzyme Q_10_, vitamin E, idebenone, EPI-743, and MitoQ are able to increase NRF2 stability/expression and to induce NQO1 activity in cells and in animal models [77,102,103,104,105,106], which is a renowned method to evaluate the functionality of NRF2 inducers [107]; (ii) the chemical characteristics of the para-benzoquinone moiety make these molecules able to react with thiol compounds [108] and with protein-contained cysteine residues [109]; (iii) in silico modeling analyses showed a potential interaction between the vitamin E and KEAP1 [110], suggesting a common behavior for molecules sharing the same biochemical structure; and (iv) in particular conditions, especially in an oxidative stress-induced environment [111,112,113,114], these compounds can act as pro-oxidants thus directly inducing NRF2 activity (i.e., by interacting with KEAP1) or increasing the amount of oxidative species in the cell. 

Although coenzyme Q_10_, vitamin E and idebenone poorly cross the blood–brain barrier [115,116,117], these compounds have been widely tested in FA clinical trials. The co-administration of coenzyme Q_10_ and vitamin E was tested in several small trials, demonstrating a general improvement in cardiac and skeletal muscle bioenergetics, although the neurologic outcome was unaffected [118,119,120]. Contrasting results come from several studies investigating idebenone, where some promising results have been obtained on cardiac defects [121,122,123,124,125,126], while other evidence has failed to demonstrate the efficacy of idebenone in FA [127,128,129,130]. On the other hand, both MitoQ and EPI-743 display an enhanced permeability of blood–brain barrier [131,132] and are used in a wide range of diseases in which oxidative stress represents a common thread [133]. EPI-743, in particular, was used in a clinical trial where 14 patients with mitochondrial diseases (one of them was FA) were enrolled. This study showed improvements in the pathologic condition and in quality of life [131]. Progress in the Fredreich’s Ataxia Rating Scale (FARS) following EPI-743 administration was also reported in a 6-month-long phase II trial, followed by an 18-month-long open label phase and in an open-label study of G130V point mutation FA patients [134,135]. 

Resveratrol, a non-flavonoid polyphenolic antioxidant compound present in fruits, especially in red grapes [136], has been found to mediate ARE genes’ transcription by enhancing NRF2 stability and mRNA expression, thus acting in a similar way to the flavonoid quercetin [137,138,139]. Importantly, the resveratrol treatment was able to induce the transcription of a stably transfected frataxin-Green Fluorescent Protein (GFP) reporter in HeLa cells and to increase the frataxin protein and mRNA transcript in a FA mouse model and in patients’ fibroblasts and lymphoblasts [140]. Although these results could not be reproduced in peripheral blood mononuclear cells (PBMC) of FA patients, the FARS score was, however, improved in an open-label study of FA patients treated with a high dose of resveratrol [141].

Among NRF2 modulators, the triterpenoids are highly specific and extremely potent NRF2 inducers [142,143], particularly able to penetrate in the central nervous system [144]. One of these chemical compounds, RTA408, was shown to inhibit the KEAP1-mediated NRF2 degradation by interacting with KEAP1 Cys^151^. Through the activation of NRF2-mediated signaling, RTA408 induced the increase of glutathione and ATP synthesis in an in vitro model of seizure-like activity and reduced the frequency of late spontaneous seizures in status epilepticus-induced mice [144]. Notably, when tested on FA fibroblasts and neurons, RTA408 was found to prevent the mitochondrial complex I inhibition, lipid peroxidation and H_2_O_2_-induced cell death [55]. A phase II trial was set up to evaluate the effects of RTA408 in the treatment of FA (MOXIe) and the first promising results have been published demonstrating improvements in the neurological outcome. In the trial, 69 FA patients were selected to receive a placebo or RTA408 in doses of between 5–300 mg, with a daily administration over 12 weeks. An increase of 3.8 points versus baseline in modified (m)FARS score was observed in the 160 mg RTA408-treated group of patients, while a 2.3 points increase was found in the placebo group [145]. Moreover, although the primary objective of the study (i.e., peak workload during maximal exercise testing) was not improved, an increase of NRF2 target gene expression was observed in patients [145], thus the second part of the MOXIe study, with 150 mg/day dosage, is actually on-going.

## 4. NRF2 Signaling Network as a Biomarker in the Evaluation of FA Therapy Outcome

**Figure 2:**
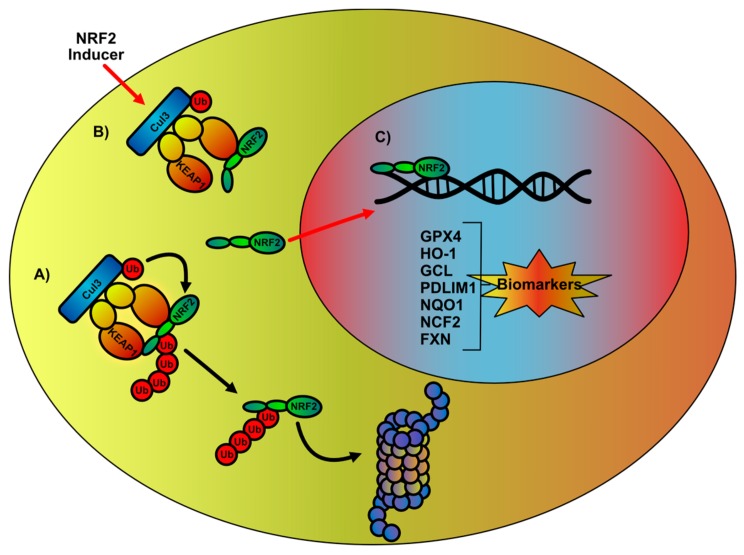
Regulation of NF-E2 p45-related factor 2 (NRF2) transcription pathway and evaluation of potential biomarkers in Friedreich’s ataxia. (**A**) In normal conditions, NRF2 is located in the cytoplasm and complexed to a Kelch-like ECH-associated protein 1 (KEAP1) homodimer, which in conjunction with Cullin3 (CUL3) determines the sequential addition of ubiquitin (Ub) monomers, leading to NRF2 polyubiquitination and proteasome-mediated degradation. (**B**) Upon ROS and/or specific compounds mediated induction, conformational changes in KEAP1 structure disrupt KEAP1 interaction with the NRF2 Neh2 domain DLG motif, impairing NRF2 polyubiquitination. This allows newly synthesized NRF2 to escape the degradation process and to migrate into the nucleus. (**C**) In the nucleus, NRF2 induces the antioxidant responsive element (ARE)-dependent transcription of specific genes whose expression significantly ameliorates the cellular defects.

Currently, there are no approved therapies for treating FA. Nevertheless, more than 20 molecules have been tested in clinics, leading in some cases to improvement in patients’ living conditions and disease progression [146,147]. It is important to note that typical clinical trials in FA consist of small pilots, with some larger follow-up studies, which often do not reach 12 months in duration [148]. This is a central point to take in account in treatment, because the clinical endpoints actually in use in FA, such as the 25-foot walk, 9-hole peg test, FARS or Scale for the Assessment and Rating of Ataxia (SARA), need to be analyzed after longer periods of time [148,149,150,151,152]. In addition, it has to be considered that after the molecular diagnosis of the GAA repeat length, a number of intermediate steps assessing the efficacy of the treatment in slowing the FA progression and/or reversing symptoms should further be evaluated. This becomes crucial to understand if a potential drug may be a promising therapeutic tool in FA and to allow a faster development of new effective therapies. To this end, the frataxin expression analysis represents a natural candidate, as it constitutes the main and the triggering cause of the disease. Nevertheless, as frataxin depletion also determines ROS increase and dysregulation of antioxidant responses [42,56,57,58,59] and promising results come by using antioxidants and NRF2 inducers in experimental models and in clinics, we believe that monitoring NRF2 and its signaling pathway in patients during treatment could provide a low-invasive and quantifiable method to evaluate the effect of ongoing therapies. In this context, a work from Hayashi and Cortopassi analyzed the expression of 84 genes involved in ROS response in FA patient-derived lymphoblasts. The authors also correlated the expression of these genes with frataxin levels and their modulation following treatment with the NRF2 inducer dimethyl fumarate (DMF) and type 1 histone deacetylase inhibitor (HDACi) and proposed the neutrophil cytosolic factor 2 (*NCF2*) and the PDZ and LIM domain 1 (*PDLIM1*) gene expression as biomarkers in FA to assess drug effectiveness [153]. NCF2 is the cytosolic subunit of the multi-protein NADPH oxidase complex [154], while PDLIM1 (also known as mystique or CLP36) is a nuclear protein involved in the regulation of NF-κB activity [155]. Importantly, NRF2 activity is able to regulate the expression of NCF2 [156,157] and PDLIM1 [158], with the second acquiring particular relevance as it is considered a biomarker of cardiomyocyte oxidative status (153) and participates in the regenerative process of peripheral neurons of the dorsal root ganglia [159]. Thus, monitoring PDLIM1 expression in blood could provide clues on the effectiveness of treatments in the two tissues mostly affected in FA. Furthermore, we have recently demonstrated a significant impairment of the expression of prototypic NRF2-regulated genes in FA fibroblasts and their re-establishment after NRF2 induction [77]. In particular, the efficacy of six redox-active drugs (i.e., idebenone, EPI-743, RTA408, N-acetyl cysteine (NAC), DMF and SFN) have been compared on the expression of NRF2 and its target genes (NAD(P)H, quinone dehydrogenase 1 (NQO1) heme oxygenase 1, HO-1 and glutamate cysteine ligase (GCL)). All drugs tested consistently increased NRF2 expression and, depending on the molecule, the expression of at least one of the assessed NRF2 target genes was recovered by treatments [77]. Glutathione (GSH) cellular content was also restored after NRF2 induction [77]. As both genes responsible of glutathione synthesis (*glutathione synthetase (GSS)* and *gamma-glutamyl cysteine synthase (GCL)*) are under control of NRF2 activity [160,161], this panel of genes together with the GSH cellular content can be assessed in blood of patients, discriminating the pathologic state in FA (as in other neurodegenerative conditions [162]) and providing appropriate feedbacks on the therapeutic interventions (Figure 2). Importantly, evolution-conserved ARE sequences are present in the *FNX* gene and dyclonine-mediated NRF2 induction [163] as well as resveratrol treatment [140], are able to increase frataxin expression. The increase of *FXN* mRNA was also demonstrated in FA fibroblasts treated with different NRF2 inducers [77], thus strongly suggesting that intervening in the restoration of the FA-induced defects on NRF2 molecular circuitry could lead to the rescue of FA primary defect, (i.e., frataxin protein deregulation), as well as to re-balance the secondary hit (the increased oxidative stress). Until now, NRF2-induced changes in frataxinexpression were modest and inducers were able to increase frataxinin specific cell types but not in others [140,141]. This allows some speculations: (i) the number and the sequence of ARE elements may vary depending on the gene [164], suggesting that some ARE elements can be stronger than others, with specific genes preferably transcribed depending on the magnitude of the NRF2 activation; (ii) several NRF2 co-transcriptional factors participate in the NRF2-mediated gene regulation [63,64,165], highlighting that cellular-specific differential expression of the transcriptional regulatory machinery could determine alternative results; and (iii) different profiles of gene expression have been obtained by diverse NRF2 inducers [77], thus suggesting a specific pattern of activation for its signaling pathway and paving the way for the synthesis of new molecules and the set-up of combined treatments aimed at obtaining discrete outcomes. 

## 5. Conclusions

FA is a neurodegenerative disorder for which no cure is actually at our disposal. Due to the ubiquitous nature of the frataxin expression, FA is a multisystemic disease where, despite the death of dorsal root ganglia (DRG) neurons and cardiomyopathy, which represent, respectively, the first pathologic event and the primary cause of death [21,146], patients also display a number of secondary defects including diabetes, hearing loss, visual impairments, and cognitive deficits [146]. As impairments in the main pathologic targets can be reflected in other cellular districts, even less affected (i.e., blood), this suggests that the identification of sensitive and reliable biomarkers in FA could be useful in evaluating both the effectiveness of specific therapies and in monitoring the disease progression. Most of the standard clinical outcomes in FA need at least one year to be reliable and this slows drug development and makes necessary the setup of long clinical trials [148]. Since much genetic and biochemical evidence has attested to alterations of NRF2 signaling in FA [56,57,58,59], here we propose monitoring the NRF2 pathway in the steps preceding the endpoints of clinical trials, in order to speed up the identification of effective drugs, allowing the selection and/or synthesis of new and more potent compounds. Of note, NRF2 inducers are able to determine diverse outcomes in the panel of genes and biochemical effectors (e.g., GSH levels) that can be used to assess the efficacy of a therapeutic drug in FA, thus also opening the way to the development of therapies based on the combination of molecules aimed at obtaining optimal results.
